# Time until Need for Levodopa among New Users of Dopamine Agonists or MAO-B Inhibitors

**DOI:** 10.1155/2021/9952743

**Published:** 2021-07-01

**Authors:** Caroline D. Binde, Ingunn F. Tvete, Marianne Klemp

**Affiliations:** ^1^Department of Pharmacology, University of Oslo, Oslo, Norway; ^2^Norwegian Computing Center, Oslo, Norway

## Abstract

**Objective:**

To investigate the use of dopamine agonists and monoamine oxidase type B (MAO-B) inhibitors in the Norwegian population, between 1 July 2006 and 31 December 2016. Our primary endpoint was time until need for levodopa among new monotherapy users of dopamine agonists and MAO-B inhibitors.

**Methods:**

A prospective cohort study including all patients, aged 50 years or above, who had at least one prescription for a dopamine agonist or a MAO-B inhibitor dispensed in the study period. We used data from the Norwegian Prescription Database (NorPD). As we wished to focus on new Parkinson patients, we excluded patients who had levodopa dispensed less than 180 days prior to their first dopamine agonist or MAO-B inhibitor redemption. We explored the demographics and the time until monotherapy was insufficient treatment (defined as need for levodopa prescription).

**Results:**

We included 22958 new monotherapy users. Of these, 22108 used dopamine agonists and 850 used MAO-B inhibitors. The mean number of days until the first prescription of levodopa was dispensed was higher among the dopamine agonist users (621 days) compared to the MAO-B inhibitor users (352 days). The proportion of dopamine agonist users who started levodopa treatment during the study period was less than 7%, while the corresponding proportion of MAO-B inhibitor users was almost 59%.

**Conclusions:**

We found that new dopamine agonist users had a much greater delay in the need for levodopa than new MAO-B inhibitor users. It seems to be beneficial to initiate treatment with dopamine agonists when starting pharmacological treatment for new Parkinson patients.

## 1. Introduction

Parkinson's disease is a progressive neurodegenerative disorder, associated with continuing loss of dopaminergic neurons in the substantia nigra [1]. Patients with Parkinson's disease develop motor symptoms such as tremor, rigidity, and bradykinesia [1]. Symptomatic treatment of Parkinson's disease is based on the replacement of dopamine, mainly via the dopamine precursor levodopa [1, 2]. Managing the symptoms of Parkinson's disease can be a challenging task, as chronic levodopa treatment is associated with development of side effects, such as motor symptoms and dyskinesia [1, 2]. Identifying effective alternatives to levodopa could postpone the introduction of levodopa treatment and hence delay the development of levodopa side effects. This could be particularly important for younger patients, where delaying the onset of levodopa side effects could enable them to keep working and have a better quality of life. Furthermore, the ongoing debate regarding the possible neurotoxicity of levodopa [3] adds motive to delay or reduce the use of levodopa where effective alternatives are available.

Several agents are available for symptomatic treatment of Parkinson's disease, and monoamine oxidase type B (MAO-B) inhibitors and dopamine agonists are available as adjuncts or alternatives to levodopa [4]. To delay the use of levodopa and the development of side effects, starting treatment with one of these agents could be preferred for newly diagnosed patients. As treatment guidelines recommend these agents [4, 5], comparing the actual use of these two alternatives in a real-life setting can help establish which initial treatment is preferable with respect to delaying the need for levodopa. It will also provide valuable information for optimizing anti-Parkinson treatment and can be part of treatment guidelines.

We have previously investigated the comparative effectiveness of three MAO-B inhibitors and four dopamine agonists used in Parkinson's disease, in two multiple treatment comparison (MTC) network meta-analyses [6, 7]. We found that all drugs included (except safinamide) were effective as monotherapy for Parkinson's disease; however, the dopamine agonists, pramipexole and ropinirole, ranked higher than the MAO-B inhibitors [6, 7]. We have extended this research by studying the actual use of these drugs in real life.

Others have previously investigated the prescribing patterns and use of anti-Parkinson medications in the USA [8], Europe [9, 10], and Australia [11]. These studies were conducted by assessing data from inpatient hospital stays [8], medical records [10], outpatient sales statistics [9], and use of outpatient prescribed drugs [11]. To add to this knowledge, we present the results from a prospective cohort study involving individual patient level data regarding patients receiving either MAO-B inhibitors or dopamine agonists in the Norwegian population, using data from the Norwegian Prescription Database (NorPD). Using data from the prescription database reduces the risk of recall and selection bias and adds valuable information regarding the drugs' effectiveness [12, 13]. Knowledge obtained from observational register studies add important insight and help establish an effective treatment strategy for Parkinson's disease.

## 2. Patients and Methods

### 2.1. Study Design

This is a prospective cohort study involving patients with at least one MAO-B inhibitor or dopamine agonist prescription dispensed from a Norwegian pharmacy used as monotherapy during an eleven-year period. The Regional Committees for Medical and Health Research Ethics of Southeast Norway (REK) approved this study (2017/1833), and the project is registered with Current Research Information System in Norway [14].

### 2.2. Data Sources

We obtained data from the Norwegian Prescription Database (NorPD). NorPD is a nationwide registry, automatically collecting data on all drugs dispensed from Norwegian pharmacies [12, 13, 15]. The drugs were classified according to the ATC index [16]. Every person born in or immigrating to Norway is given a unique national identity number [12, 17]. This unique identity number makes it possible to collect data at an individual patient level and is a valuable and reliable source to study the actual drug use in a real-life setting [12, 13].

### 2.3. Study Population

**Figure 1:**
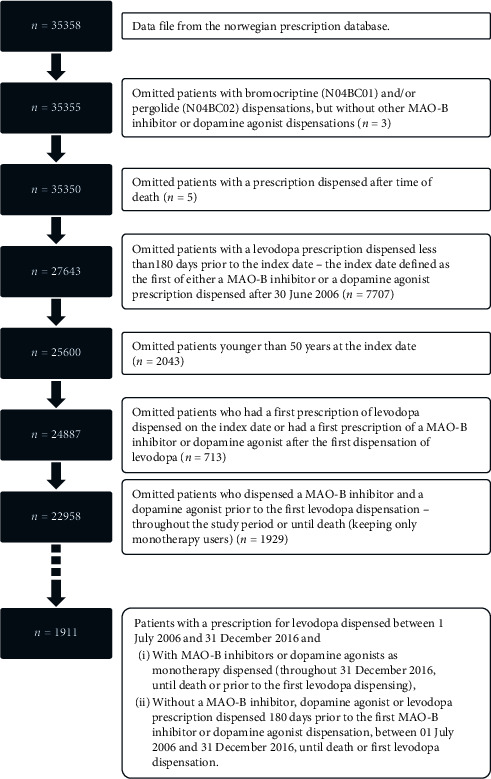
Overview of the data handling procedure.

We considered data on all patients, aged 50 years or above, who had at least one prescription dispensed from a Norwegian pharmacy for MAO-B inhibitors (ATC N04BD01, selegiline; N04BD02, rasagiline; or N04BD03, safinamide) or dopamine agonists (ATC N04BC06, cabergoline; N04BC05, pramipexole; N04BC09, rotigotine; or N04BC04, ropinirole) between 1 January 2006 and 31 December 2016. We followed the patients from the first dispensing date of either a MAO-B inhibitor or a dopamine agonist, defined as the index date. As we sought to follow new monotherapy users, we excluded patients who had a prescription for MAO-B inhibitors, dopamine agonists, or levodopa dispensed during the 180 days prior to the index date, making 1 July 2006 the first possible index date. We followed them until they reached one of the study endpoints: dispensing levodopa, death, or throughout 2016 (end of study period). We only considered patients using either MAO-B inhibitors or dopamine agonists as monotherapy. We present the data handling procedure in Figure 1.

### 2.4. Baseline Assessments from NorPD

We retrieved data on the prescriber's specialty, the patients' age and gender, and whether they had prescriptions for drugs used in diabetes, thyroid hormones, antihypertensive, or antithrombotic drugs dispensed prior to the index date. We used this information as an indication of types and severity of comorbidity, drugs used in diabetes as an indication of diabetes, thyroid hormones as an indication of hypothyroidism, antihypertensive drugs as an indication of hypertension, and antithrombotic agents as an indication of atrial fibrillation.

### 2.5. Outcomes

The primary objective of this analysis was to compare the time until need for levodopa, defined as the first dispensing date of levodopa, among new monotherapy users of MAO-B inhibitors and dopamine agonists. We compared these two groups of patients taking into consideration the patients' age, gender, the specialty of the first prescriber of MAO-B inhibitors or dopamine agonists, and the patients' comorbidities (background information).

### 2.6. Statistical Analyses

We conducted the statistical analyses in the open source statistical software R [18]. We drew Kaplan–Meier plots to explore the time until need for levodopa for men versus women, for MAO-B inhibitor versus dopamine agonists users, for diabetic versus nondiabetic patients, for patients with and without hypothyroidism, for patients with and without hypertension, for patients with and without atrial fibrillation, and for patients with a first prescription given by a specialist versus a nonspecialist.

**Figure 2:**
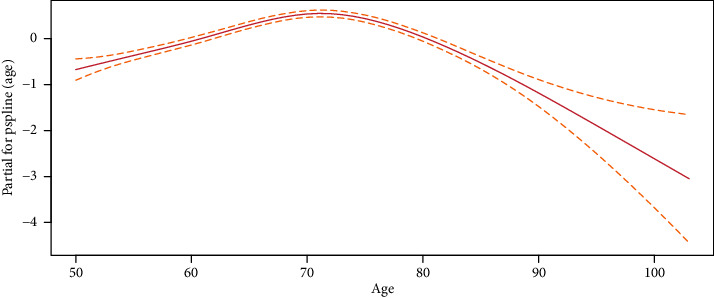
Estimated age-response curve: the risk of age on time to levodopa onset.

We first considered time until need for levodopa as a function of age by doing a Cox regression analysis by interpolation (so-called splines), as shown in Figure 2. This way, we let the time until need for levodopa depend upon age in a nonlinear way, and this revealed a linear relationship between age and time until need for levodopa for ages below 72 years (hereafter, referred to as the younger age group), and another linear relationship for ages 72 years and above (hereafter, referred to as the elder age group). Following this, we decided to conduct one analysis for the younger group and another for the elder group.

For these two age groups, we conducted Cox regression analyses for the time until need for levodopa given the background information. We considered one analysis for the time until need for levodopa taking into consideration each of the background variables and one analysis including all the background variables simultaneously. We then found an optimal model with respect to which of the background variables were relevant by using an automatic model selection procedure based on Akaike's information criterion [19].

## 3. Results

We found 22958 patients who had at least one prescription for a MAO-B inhibitor or a dopamine agonist dispensed between 1 July 2006 and 31 December 2016, used as monotherapy. Of these, 850 had prescriptions for MAO-B inhibitors and 22108 for dopamine agonists.  Table 1 presents patient characteristics for these groups. We found the proportion of males to be 58% in the MAO-B inhibitor group and 38% in the dopamine agonist group. The average number of days until need for levodopa was 352 days in the MAO-B inhibitor group and 621 days in the dopamine agonist group. The corresponding median days were 187 days in the MAO-B inhibitor group and 315 days in the dopamine agonist group. The proportion of patients who had the first prescription prescribed by a specialist was 3.9% in the MAO-B inhibitor group and 1% in the dopamine agonist group.

Among the 22958 new monotherapy users, 1911 redeemed at least one prescription for levodopa during the study period and 21047 did not.  Table 2 presents the patient characteristics for these two groups. The proportion of males was 55% in the group who had levodopa dispensed and 38% in the group who did not have levodopa dispensed. The proportion of patients who had levodopa dispensed was almost 59% in the MAO-B inhibitor group and almost 7% in the dopamine agonist group.

When considering age in relation to the time until need for levodopa, we revealed a linear relationship between age and time until need for levodopa for the age group below 72 years of age, and a another linear relationship for the age group of 72 years or above (Figure 2). We therefore considered time until need for levodopa separately for the two age groups, and the patient characteristics for each of these groups are listed in Tables S2 and S3 in the Supplementary Materials. Tables 3 and 4 present the results from the Cox proportional hazard regression analyses (final model) for these two groups.

We found that for both age groups, women had a lower risk of starting levodopa treatment than men (HR = 0.609, CI = (0.541, 0.685) for the younger group and HR = 0.609, CI =(0.523, 0.708) for the older group). When considering the age group below 72 years, we found that the risk of having a levodopa prescription dispensed increased with age (HR = 1.054, CI = (1.043, 1.065)). For the older group, we found the risk of having a levodopa prescription dispensed decreased with age (HR = 0.924, CI = (0.91, 0.938)). For the younger group, we found that those who had their first prescription of a MAO-B inhibitor or dopamine agonist prescribed by a specialist had two times the higher risk of having a levodopa prescription dispensed than those who did not have their first prescription prescribed by a specialist (HR = 2.003, CI = (1.446, 2.775)). In the final model presented in Table 4, we see that the variable “first prescriber's specialty” was not part of the model. Considering a model only regressing on the first prescriber's specialty, this factor was significant (HR = 1.994, CI = (1.127, 3.527)), but in a full model considering all variables, it was not significant (HR = 0.798, CI = (0.448, 1.421). In both age groups, the risk of initiating levodopa treatment was lower for dopamine agonist users compared to MAO-B inhibitor users (HR = 0.101, CI = (0.088, 0.118) for the younger group and HR = 0.064, CI = (0.055, 0.075) for the older group).

## 4. Discussion

The results from this prospective cohort study indicate that, among new monotherapy users of MAO-B inhibitors and dopamine agonists, dopamine agonists might postpone the need for levodopa. Our main outcome was time until need for levodopa, which we defined as the first dispensing date of levodopa in the study period. We found that new monotherapy users of MAO-B inhibitors had a prescription for levodopa dispensed on average almost a year earlier than new monotherapy users of dopamine agonists, and eventually, a much larger proportion of the MAO-B inhibitor users ended up dispensing levodopa. We notice that the median days until redeeming levodopa was almost half the number of average days, indicating skewed distributions of days until the first levodopa redemption. Hence, some patients had a very long period from initiation of the MAO-B inhibitor or dopamine agonist treatment until levodopa redemptions. This contributed to the high average number of days.

We note that the dopamine agonist group was considerably larger (*n* = 22 108) than the MAO-B inhibitor group (*n* = 850). This difference is interesting as it may reflect the prescriber's preferences when initiating anti-Parkinson therapy. However, from this analysis, we have no explanation to why there is a difference, and it would be interesting to explore this in further research.

Overall, relatively few patients received their first prescription from a specialist. Still, a much greater fraction of the MAO-B inhibitor users received their first prescription from a specialist compared to the dopamine agonist users. Considering the comedication variables for the two user groups, as presented in Table 1, we cannot see any plausible explanations to this difference. A possible explanation could be that patients in the MAO-B inhibitor group had easier access to specialist care compared to patients in the dopamine agonist group. In this regard, it could have been interesting to look at the geographical differences between these groups. Norway has a relatively widespread decentralized population covering a large area, with easier access to specialist care for those living closer to a big city than in more rural areas [20].

Levodopa is still considered the gold standard for symptomatic treatment of Parkinson's disease [21], and as the disease progresses, most patients will ultimately need levodopa treatment [1]. However, considering the development of levodopa side effects, it would be in the best interest of the patient to delay the initiation of levodopa treatment, and hence, the development of side effects. Treatment recommendations and guidelines offer a choice from a range of options, but do not clearly identify a preferred treatment strategy for new monotherapy users among patients with Parkinson's disease [4, 5]. We have previously investigated the comparative effectiveness of four dopamine agonists (pramipexole, ropinirole, rotigotine, and cabergoline) and three MAO-B inhibitors (selegiline, rasagiline, and safinamide) in two multiple treatment comparison (MTC) meta-analyses [6, 7]. In these studies, we found that both MAO-B inhibitors and dopamine agonists are effective and safe as monotherapy (except safinamide) for patients with Parkinson's disease, and we found the dopamine agonists ropinirole and pramipexole to be most effective among these drugs, compared to placebo [7]. Our current results are in line with these findings on a drug class level. A noninterventional, observational study assessing the efficacy and safety of several dopamine replacement therapies in a real-life setting observed a beneficial effect of dopamine agonists both when given alone and in combination with levodopa [22]. Our results reflect these findings to a certain extent. In addition, they reported a decrease in the proportion of patients who received dopamine agonists as monotherapy and an increase in the proportion of patients who received combination therapy with dopamine agonists and levodopa during the study period [22]. These findings support the theory that, in time, most patients will need combination therapy [22].

When considering how age relates to the risk of starting levodopa treatment, we divided the patients in two age groups, as the relationship changed around the age of 72. For the group below 72 years, we found that the risk of starting levodopa treatment increased with age. For the group aged 72 years and above, the risk of starting levodopa treatment decreased with age. This latter finding was perhaps somewhat surprising as we expected to see the need for levodopa treatment increase with age for all ages. A possible explanation might be that a larger fraction of the older patients died before they reached the endpoint “need for levodopa” and that the risk of initiating levodopa treatment therefore decreased with higher age.

When looking at time until need for levodopa across genders, we found that women in both age groups had a lower risk of starting levodopa treatment than men. This could indicate better medication compliance among women or that women tend to visit their doctor more often than men do and hence are more closely followed-up.

We also considered how comedication for a set of other diseases relates to the risk of starting levodopa treatment (Table S1). Interestingly, we found that patients already treated with antidiabetic drugs had a lower risk of starting levodopa treatment. A possible explanation for this could be that these patients are used to following a treatment plan and, therefore, more compliant to treatment plans for other diseases, such as Parkinson's disease. In relation to this, one could also imagine that these patients are regularly in contact with their doctor and therefore more closely monitored.

We recognise that there are both advantages and limitations to this study. The data obtained from the NorPD are on an individual patient level and collected automatically to the prescription database [15]. This eliminates the risk of recall and selection bias. As a population-based analysis, there was no observational bias. The data does not include information regarding treatment indication or the patients' diagnosis, and we cannot assess the patients' compliance. The drugs included in our study are indicated for treatment of Parkinson's disease, although we cannot exclude the possibility that some patients included in this analysis used these drugs for other purposes than Parkinson's disease; however, we find it unlikely that the use for other purposes would be substantial.

We defined new MAO-B inhibitor and dopamine agonist users as patients without redemptions of levodopa for at least 6 months prior to the first MAO-B or dopamine agonist redemption. Since Parkinson's disease is a chronic, noncurable disease, we consider 6 months without any anti-Parkinson drug use as sufficient to define new users. We have not considered the amount dispensed or the frequency of dispensations of MAO-B inhibitors or dopamine agonists throughout the observation period. Possibly, some dispensed higher doses and/or more frequently than others. We have also not considered whether the MAO-B inhibitor and dopamine agonist users switched drugs within the drug class during the observation period. This could give a more detailed and complex analysis but would likely result in subanalyses from small subcohorts.

## 5. Conclusions

In conclusion, the results from this analysis show that new dopamine agonist users had a much greater delay in the need for levodopa than new MAO-B inhibitor users, which may indicate that it can be beneficial to start anti-Parkinson monotherapy with a dopamine agonist in newly diagnosed Parkinson patients.

## Figures and Tables

**Table 1:** Patient characteristics in the MAO-B inhibitor and dopamine agonist group.

	MAO-B inhibitors (*n* = 850)	Dopamine agonists (*n* = 22108)
Proportion of men	58.12	38.23
Age distribution^*∗*^	9.41, 35.53, 39.06, 16	24.07, 30.03, 26.66, 19.24
Proportion with drugs for diabetes (none)	6.12	9.98
Proportion with drugs for hypothyroidism (none)	8.71	12.76
Proportion with drugs for hypertension (none)	11.53	15
Proportion with drugs for atrial fibrillation (none)	42.12	39.37
Proportion with first prescription for the MAO-B inhibitor/dopamine agonist from the specialist	3.88	1
Days observed^∗∗^	712, 362, 1, 3789	1733, 1623.5, 1, 3836
Proportion with levodopa prescription dispensed	58.59	6.39
Days until levodopa prescription dispensed^∗∗^	352, 187, 1, 2081	621, 315, 1, 3665

^*∗*^Age groups, 50–59, 60–69, 70–79, and 80+. ^∗∗^Mean, median, min, and max.

**Table 2:** Patient characteristics in the group with levodopa dispensation and with no levodopa dispensation.

	No levodopa redemption (*n* = 21047)	Levodopa redemption (*n* = 1911)
Proportion of men	37.55	54.47
Age distribution^*∗*^	24.19, 29.7, 26.2, 19.9	16.22, 36.05, 37.21, 10.52
Proportion with drugs for diabetes (none)	10.09	7.01
Proportion with drugs for hypothyroidism (none)	12.85	10.05
Proportion with drugs for hypertension (none)	15.14	11.93
Proportion with drugs for atrial fibrillation (none)	39.61	37.94
Proportion with first prescription for the MAO-B inhibitor/dopamine agonist from the specialist	0.97	2.62
Days observed^∗∗^	1800, 1717, 1, 3836	551, 277, 1, 3665

^*∗*^Age groups, 50–59, 60–69, 70–79, and 80+. ^∗∗^Mean, median, min, and max.

**Table 3:** Cox regression analyses: time until first dispensing date of levodopa for patients under 72 years, the final model (baseline in parentheses).

Variable	HR	CI	*P* value
Age	1.054	(1.043, 1.065)	<0.001
Gender (male)	0.609	(0.541, 0.685)	<0.001
Drugs for diabetes (none)	0.824	(0.659, 1.03)	0.089
Drugs for hypertension (none)	0.816	(0.664, 1.003)	0.053
Drugs for atrial fibrillation (none)	0.888	(0.777, 1.014)	0.080
1^st^ prescription from specialist (not specialist)	2.003	(1.446, 2.775)	<0.001
Dopamine agonist user (MAO-B inhibitor user)	0.101	(0.088, 0.118)	<0.001

**Table 4:** Cox regression analyses: time until first dispensing date of levodopa for patients 72 years or above, the final model (baseline in parentheses).

Variable	HR	CI	*P* value
Age	0.924	(0.91, 0.938)	<0.001
Gender (male)	0.609	(0.523, 0.708)	<0.001
Drugs for diabetes (none)	0.693	(0.513, 0.935)	0.016
Drugs for hypothyroidism (none)	1.207	(0.963, 1.513)	0.102
Dopamine agonist user (MAO-B inhibitor user)	0.064	(0.055, 0.075)	<0.001

## Data Availability

The data used to support the findings of this study were supplied by the Norwegian Prescription Database and cannot be made freely available by the authors. Application for access to these data can be made by anyone to the Norwegian Institute of Public Health.

## Supplementary Materials

Supplementary Materials Table S1. Variables obtained from NorPD. Table S2. Patient characteristics of the groups with and without levodopa redemption below 72 years of age. Table S3. Patient characteristics of the groups with and without levodopa redemption, aged 72 years or above.

## References

[1] Goetz C. G., Pal G. (2014). Initial management of Parkinson’s disease. *BMJ*.

[2] Rascol O., Lozano A., Stern M., Poewe W. (2011). Milestones in Parkinson’s disease therapeutics. *Movement Disorders*.

[3] Müller T. (2020). Pharmacokinetics and pharmacodynamics of levodopa/carbidopa cotherapies for Parkinson’s disease. *Expert Opinion on Drug Metabolism & Toxicology*.

[4] Fox S. H., Katzenschlager R., Lim S. Y. (2018). International parkinson and movement disorder society evidence-based medicine review: update on treatments for the motor symptoms of parkinson’s disease. *Movement Disorder*.

[5] National Institue for Health and Care Exellence (2017). *Parkinson’s Disease in Adults*.

[6] Binde C. D., Tvete I. F., Gåsemyr J., Natvig B., Klemp M. (2018). A multiple treatment comparison meta-analysis of monoamine oxidase type B inhibitors for Parkinson’s disease. *British Journal of Clinical Pharmacology*.

[7] Binde C. D., Tvete I. F., Gåsemyr J. I., Natvig B., Klemp M. (2020). Comparative effectiveness of dopamine agonists and monoamine oxidase type-B inhibitors for Parkinson’s disease: a multiple treatment comparison meta-analysis. *European Journal of Clinical Pharmacology*.

[8] Crispo J. A. G., Fortin Y., Thibault D. P. (2015). Trends in inpatient antiparkinson drug use in the USA, 2001-2012. *European Journal of Clinical Pharmacology*.

[9] Rosa M. M., Ferreira J. J., Coelho M., Freire R., Sampaio C. (2010). Prescribing patterns of antiparkinsonian agents in Europe. *Movement Disorders*.

[10] Trifirò G., Savica R., Morgante L. (2008). Prescribing pattern of anti-Parkinson drugs in Southern Italy: cross-sectional analysis in the years 2003-2005. *Parkinsonism & Related Disorders*.

[11] Hollingworth S. A., Rush A., Hall W. D., Eadie M. J. (2011). Utilization of anti-Parkinson drugs in Australia: 1995-2009. *Pharmacoepidemiology and Drug Safety*.

[12] Furu K., Wettermark B. r., Andersen M., Martikainen J. E., Almarsdottir A. B., SÃ¸rensen H. T. (2010). The Nordic countries as a cohort for pharmacoepidemiological research. *Basic & Clinical Pharmacology & Toxicology*.

[13] Wettermark B., Zoëga H., Furu K. (2013). The Nordic prescription databases as a resource for pharmacoepidemiological research-a literature review. *Pharmacoepidemiology and Drug Safety*.

[14] Current Research Information System (2017). *Norway. Legemidler Til Pasienter Med Parkinson’s Sykdom*.

[15] Berg C., Olsen K., Sakshaug S. (2019). *The Norwegian Prescription Database 2014–2018*.

[16] WHO Collaborating Centre for Drug Statistics Methodology (2021). ATC Classification index with DDDs. https://www.whocc.no/atc_ddd_index/.

[17] The Norwegian Tax Administration National identity number. https://www.skatteetaten.no/en/person/foreign/norwegian-identification-number/national-identity-number/.

[18] RStudio Team (2015). RStudio: integrated development for R. http://www.rstudio.com/.

[19] Step-function i R. https://www.rdocumentation.org/packages/stats/versions/3.6.2/topics/step.

[20] Iversen T., Kopperud G. S. (2005). Regulation versus practice-the impact of accessibility on the use of specialist health care in Norway. *Health Economics*.

[21] Fabbri M., Perez-Lloret S., Rascol O. (2020). Therapeutic strategies for Parkinson’s disease: promising agents in early clinical development. *Expert Opinion on Investigational Drugs*.

[22] Müller T., Tolosa E., Badea L. (2018). An observational study of rotigotine transdermal patch and other currently prescribed therapies in patients with Parkinson’s disease. *Journal of Neural Transmission*.

