# Supplementary material for: Time until Need for Levodopa among New Users of Dopamine Agonists or MAO-B Inhibitors
Source: Parkinsons Dis. 2021 Jul 1;2021:9952743. doi: 10.1155/2021/9952743 (PMC8270692; doi:10.1155/2021/9952743)
Supplement: Supplementary Materials — Table S1. Variables obtained from NorPD. Table S2. Patient characteristics of the groups with and without levodopa redemption below 72 years of age. Table S3. Patient characteristics of the groups with and without levodopa redemption, aged 72 years or above. [file 9952743.f1.docx]

**Supplementary materials**

**Table S1:** Variables obtained from NorPD

| *Variable* | *Group* | *ATC code* |
| --- | --- | --- |
| Age | 50-59, 60-69, 70-79, 80 years and above |  |
| Gender | Men and women |  |
| Drugs used before the index date | Drugs used in diabetes  Thyroid hormones  Antihypertensive drugs  Antithrombotic agents | A10AB, A10BA  H03AA01  C09AA, C09BA  B01AA, B01AC06, B01AE, B01AF |
| Drugs for Parkinson’s disease | MAO-B inhibitors  Dopamine agonists  Levodopa | N04BD01, N04BD02, N04BD03  N04BC04, N04BC05, N04BC06, N04BC07, N04BC09  N04BA |
| Prescriber information | First prescriber specialist or not |  |
| Death | Year and month of death |  |

**Table S2:** Patient characteristics of the groups with and without levodopa redemption, aged below 72 years

|  | No levodopa redemption (n=12545) | Levodopa redemption (n=1170) |
| --- | --- | --- |
| Proportion of men | 39.78 | 55.56 |
| Age distribution* | 40.59, 49.84, 9.57, 0 | 26.5, 58.89, 14.62, 0 |
| Proportion with drugs for diabetes (none) | 9.88 | 7.52 |
| Proportion with drugs for hypothyroidism (none) | 11.56 | 8.63 |
| Proportion with drugs for hypertension (none) | 11.19 | 9.23 |
| Proportion with drugs for atrial fibrillation (none) | 27.99 | 28.55 |
| Proportion with first prescription for MAO-B inhibitor/dopamine agonist from specialist | 1.11 | 3.25 |
| Days observed ** | 1919 , 1905 , 1 , 3836 | 575 , 281.5 , 1 , 3665 |

*Age groups: 50-59, 60-69, 70-79, 80+

**mean, median, min, max

**Table S3:** Patient characteristics of the groups with and without levodopa redemption, aged 72 years or above

|  | No levodopa redemption (n=8502) | Levodopa redemption (n=741) |
| --- | --- | --- |
| Proprortion of men | 34.27 | 52.77 |
| Age distribution* | 0, 0, 50.73, 49.27 | 0, 0, 72.87, 27.13 |
| Proportion with drugs for diabetes (none) | 10.41 | 6.21 |
| Proportion with drugs for hypothyroidism (none) | 14.75 | 12.28 |
| Proportion with drugs for hypertension (none) | 20.96 | 16.19 |
| Proportion with drugs for atrial fibrillation (none) | 56.75 | 52.77 |
| Proportion with first prescription for MAO-B inhibitor/dopamine agonist prescribed by specialist | 0.76 | 1.62 |
| Days observed** | 1623 , 1487 , 1 , 3836 | 513 , 269 , 1 , 3583 |

*Age groups: 50-59, 60-69, 70-79, 80+

**mean, median, min, max
